# Low pH impairs complement-dependent cytotoxicity against IgG-coated target cells

**DOI:** 10.18632/oncotarget.12412

**Published:** 2016-10-03

**Authors:** Ezequiel Dantas, Fernando Erra Díaz, Pehuén Pereyra Gerber, Antonela Merlotti, Augusto Varese, Matías Ostrowski, Juan Sabatté, Jorge Geffner

**Affiliations:** ^1^ Instituto de Investigaciones Biomédicas en Retrovirus y SIDA (INBIRS), CONICET, Facultad de Medicina, Universidad de Buenos Aires, Argentina

**Keywords:** complement, pH, acidosis, cancer, rituximab

## Abstract

Local acidosis is a common feature of allergic, vascular, autoimmune, and cancer diseases. However, few studies have addressed the effect of extracellular pH on the immune response. Here, we analyzed whether low pH could modulate complement-dependent cytotoxicity (CDC) against IgG-coated cells. Using human serum as a complement source, we found that extracellular pH values of 5.5 and 6.0 strongly inhibit CDC against either B lymphoblast cell lines coated with the chimeric anti-CD20 mAb rituximab or PBMCs coated with the humanized anti-CD52 mAb alemtuzumab. Suppression of CDC by low pH was observed either in cells suspended in culture medium or in whole blood assays. Interestingly, not only CDC against IgG-coated cells, but also the activation of the complement system induced by the alternative and lectin pathways was prevented by low pH. Tumor-targeting mAbs represent one of the most successful tools for cancer therapy, however, the use of mAb monotherapy has only modest effects on solid tumors. Our present results suggest that severe acidosis, a hallmark of solid tumors, might impair complement-mediated tumor destruction directed by mAb.

## INTRODUCTION

Low values of extracellular pH are usually found in tumors and inflamed tissues. Interstitial acidification (pH 5.5–7.0) is associated with the course of inflammatory reactions against infectious agents in peripheral tissues [1–6]. Autoimmune and allergic diseases are also associated with the development of acidic microenvironments. In fact, patients with rheumatoid arthritis show low pH values in the synovial fluid of compromised joints (6.5-7.0), having the acidic pH being associated with synovial fluid leukocytosis and joint damage [7–9]. The lower airway of asthmatic patients also shows acidic values of extracellular pH during exacerbation of the disease. The pH of exhaled breath condensates from these patients is around 5.2, while healthy subjects showed values around 7.2 [10, 11]. Local acidosis is a hallmark of tumor tissues. Values of extracellular pH ranging from 5.5 to 7.0 have been described in a number of solid tumors such as brain tumors, sarcomas, breast cancer, malignant melanoma, squamous cell carcinomas, and adenocarcinomas [12–16]. This acidic environment not only promotes local invasive growth and metastasis [17, 18], but also induces multi-drug resistance due to the neutralization of weak base chemotherapeutic drugs [19].

Surprisingly, although it is widely appreciated that the immune response against pathogens, host antigens (autoimmunity) and tumor cells frequently occurs under acidic environments, not much is known about the influence of pH on the innate and the adaptive immune response. We have previously reported that low pH values induce the activation of neutrophils [20, 21] and conventional dendritic cells [22, 23], suggesting that local acidification could be recognized by immune cells as a danger-associated molecular pattern (DAMP), stimulating the immune response. These results were confirmed later in two independent studies [24, 25]. Monocyte, NK cell and T cell functions were also shown to be regulated by low pH values, in the range 6.0-7.2 [26–28]. Moreover, acidic environments have shown to delay the rate of apoptosis in neutrophils, endothelial cells and tumor cells [20, 29, 30].

The constant fragment (Fc fragment) of IgG antibodies plays a crucial role in the induction of inflammatory and cytotoxic responses mediated by humoral and cellular components of the innate immune systems. The binding of IgG antibodies to their specific antigens can lead to the activation of the complement system, the induction of antibody-dependent cellular cytotoxicity (ADCC) and the phagocytosis of opsonized targets [31]. These mechanisms play an important role in antimicrobial immunity, autoimmunity, and host response against tumors [32, 33]. Moreover, they largely explain the therapeutic effects of mAbs used in cancer immunotherapy [34, 35]. The influence of low pH on the ability of IgG antibodies to induce complement-mediated cytotoxicity (CDC) has not yet been characterized. In this study, we show that pH values similar to those found in solid tumors inhibit CDC induced by IgG antibodies.

## RESULTS

### Low pH impairs complement-dependent cytotoxicity against IgG-coated target cells

**Figure 1:**
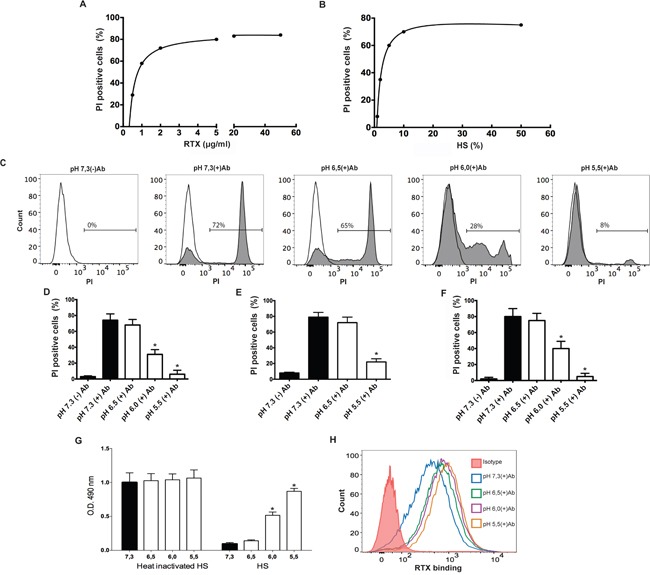
Low pH impairs CDC against RTX-coated B cell lines **A.** Raji cells (5×10^5^/100 μl) were treated with different concentrations of RTX (0.5 to 50 μg/ml) and incubated for 30 min at 37°C and pH 7.3, in RPMI medium supplemented with 10% of HS. Necrosis was then evaluated by propidium iodide staining and flow cytometry. A representative experiment (n=6) is shown. **B.** Raji cells (5 × 10^5^/100 μl) were treated with 2 μg/ml of RTX and incubated in RPMI medium supplemented with different concentrations of HS (1 to 50%) for 30 min at 37°C and pH 7.3. Then, necrosis was evaluated. A representative experiment (n=8) is shown. **C** and **D.** Raji cells (5 × 10^5^/100 μl) were treated with RTX (2 μg/ml) and then were incubated in RPMI medium supplemented with untreated (grey histograms) or heat-inactivated HS (open histograms) (10%) for 30 min at 37°C, at different values of pH. Then, necrosis was evaluated by propidium iodide staining and flow cytometry. Representative histograms and the mean ± SEM of 5 experiments performed in duplicate are shown (* p<0.05 vs pH 7.3 +Ab). **E.** Daudi cells (5 × 10^5^/100 μl) were treated with RTX (2 μg/ml) and then were incubated in RPMI medium supplemented with HS (20%) for 30 min at 37°C at different values of pH. Then, necrosis was evaluated by propidium iodide staining and flow cytometry. Data represent the mean ± SEM of 4 experiments performed in duplicate (* p<0.05 vs pH 7.3 +Ab). **F.** Raji cells (5 × 10^5^/100 μl) were treated with RTX (2 μg/ml) and then were incubated in Ca^2+^/Mg^2+^-veronal-buffered saline supplemented with 10% HS for 30 min at 37°C, at different values of pH. Then, necrosis was evaluated by propidium iodide staining and flow cytometry. Data represent the mean ± SEM of 4 experiments (* p<0.05 vs pH 7.3 +Ab). **G.** Raji cells (5 × 10^5^/100 μl) were treated with RTX (2 μg/ml) and then were incubated in RPMI medium supplemented with heat-inactivated or untreated HS (10%) for 30 min at 37°C, at different pH values. Then, cells were suspended in colourless RPMI and cell viability was analyzed using a colorimetric commercial kit based on the ability of viable cells to reduce a tetrazolium compound to a formazan product, which was quantified by absorbance at 490 nm. Data represent the mean ± SEM of 4 experiments performed in duplicate (* p<0.05 vs pH 7.3). **H.** Raji cells (5 × 10^5^/100 μl) were incubated at room temperature for 30 min with RTX (2 μg/ml) in culture medium supplemented with 10% of heat-inactivated HS adjusted to different pH values. Then, cells were washed, and the binding of RTX to Raji cells was revealed using a FITC-labeled mAb directed to the Fc fragment of human IgG and flow cytometry. A representative experiment is shown (n=4).

In a first set of experiments, we determined the concentration of the anti-CD20 chimeric antibody rituximab (RTX) needed to destroy Raji cells by complement. Using 10% HS as a complement source, we found that 2 μg/ml of RTX induces the necrosis of 60 to 75% of Raji cells after 30 min of incubation, evaluated by PI staining and flow cytometry. A representative experiment is shown in Figure 1A. Increasing the concentration of HS to 50% did not further increase cytotoxicity (Figure 1B), hence to analyze the effect of extracellular pH on cytotoxicity we used 2 μg/ml of RTX to coat Raji cells and 10% HS as a complement source.

Figures 1C and 1D show that pH 5.5 almost completely prevented CDC against Raji cells, while pH 6.0 reduced cytotoxicity by more than 50% compared with pH 7.3. No cytotoxicity was observed when HS was decomplemented by heating at 56°C for 30 min (Figure 1C). Similar results were observed using the B lymphocyte cell line Daudi instead of Raji cells (Figure 1E) or when CDC assays were performed in Ca^2+^/Mg^2+^-supplemented veronal-buffered saline instead of RPMI 1640 medium (Figure 1F). The pH-dependence of CDC was also studied using an alternative approach to evaluate cell viability, a colorimetric method, instead of PI staining, based on the reduction of the tetrazolium compound [3-(4, 5-dimethylthiazol-2-yl)-5-(3-carboxymethoxyphenyl)-2-(4-sulfophenyl)-2H-tetrazolium] to a formazan product by metabolically active cells. Consistent with the observations made by PI staining, we observed that low pH prevented Raji cell death evaluated by this alternative methodology (Figure 1G). Finally, to be sure that inhibition of CDC was not related to a diminished binding of RTX to Raji cells at low pH values, we analyzed the binding of RTX at pH 7.3, 6.5, 6.0, and 5.5. Figure 1H shows that low pH did not inhibit the binding of RTX. In fact, a significant increase in the binding of RTX to Raji cells was observed at pH 6.5, 6.0, and 5.5, compared to pH 7.3 (p<0.01, n=4).

**Figure 2:**
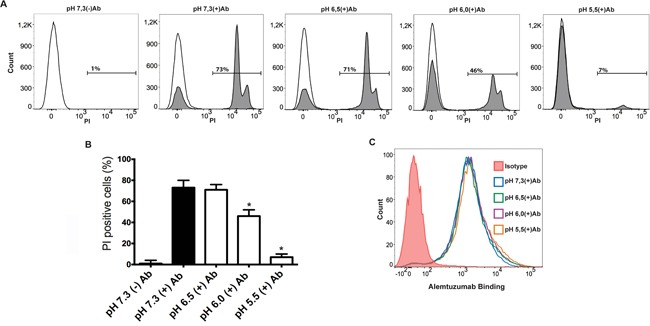
Low pH impairs CDC against alemtuzumab-coated PBMCs **A** and **B.** PBMCs (5 × 10^5^/100 μl) were treated with alemtuzumab (10 μg/ml) and then were incubated for 30 min at 37°C at distinct pH values in RPMI medium supplemented with 10% autologous HS. Necrosis was then evaluated by propidium iodide staining and flow cytometry. In (A), open histograms represent PBMCs treated with alemtuzumab (10 μg/ml) and incubated with 10% heat-inactivated autologous HS. Representative histograms (A) and the mean ± SEM of 6 experiments performed in duplicate (B) are shown (* p<0.05 vs pH 7.3). **C.** PBMCs (5 × 10^5^/100 μl) were incubated for 30 min at room temperature with alemtuzumab (10 μg/ml) in culture medium supplemented with 10% of heat-inactivated autologous HS adjusted to different values of pH. Then, cells were washed, and the binding of alemtuzumab to PBMCs was revealed using a FITC-labeled mAb directed to the Fc fragment of human IgG and flow cytometry. A representative experiment is shown (n=3).

To validate the observations made in B cell lines to other cell types, we analyzed the effect of pH on CDC against PBMCs coated with the humanized anti-CD52 mAb alemtuzumab. All these experiments were performed using autologous serum. Preliminary experiments showed that 10 μg/ml of alemtuzumab saturated the PBMC's binding capacity, evaluated by flow cytometry (data not shown). Figures 2A and 2B show that pH 5.5 almost completely impaired CDC, while pH 6.0 significantly prevented cytotoxicity. No cytotoxicity was observed when HS was decomplemented by heating at 56°C for 30 min (Figure 2A). As expected, no inhibition in the binding of alemtuzumab to PBMCs was observed in the range of pH analyzed (Figure 2C). Together, our results suggest that severe acidosis (pH 5.5 and 6.0) prevents CDC irrespective of the nature of target cells.

### Low pH impairs complement-dependent cytotoxicity against IgG-coated target cells mediated by both the classical and alternative pathways of activation

**Figure 3:**
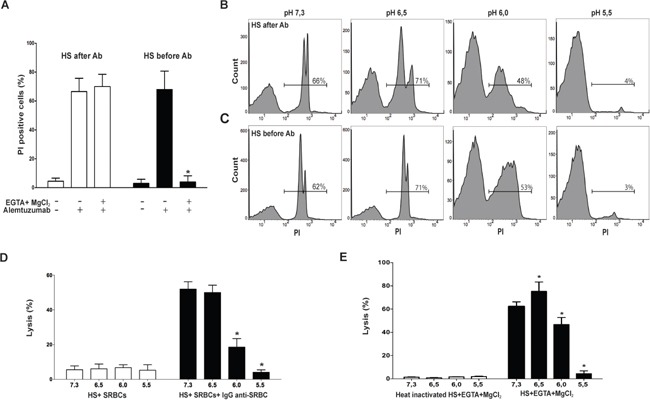
Low pH impairs CDC mediated by both the classical and alternative pathways of activation **A.** Open bars: PBMCs (5 × 10^5^/100 μl) were treated with alemtuzumab (10 μg/ml), washed and incubated for 30 min at 37°C and pH 7.3 with RPMI 1640 medium supplemented with 10% HS, in the absence or presence of 5mM MgCl_2_ and 10 mM EGTA (EGTA/Mg). Black bars: PBMCs were suspended in RPMI 1640 medium supplemented with 10% HS (5 × 10^5^/100 μl) and then were treated with alemtuzumab (10 μg/ml). Cells were incubated for 30 min at 37°C and pH 7.3, in the absence or presence of EGTA/Mg. In all cases, necrosis was evaluated by propidium iodide staining and flow cytometry. Data represent the mean ± SEM of 4 experiments performed in duplicate. * p<0.05 vs PBMCs incubated with alemtuzumab in the absence of EGTA/Mg. **B.** PBMCs (5 × 10^5^/100 μl) were treated with alemtuzumab (10 μg/ml), washed and incubated for 30 min at 37° in RPMI 1640 medium supplemented with 10% HS, at different pH values. Necrosis was then evaluated by propidium iodide staining and flow cytometry. A representative experiment (n=4) is shown. **C.** PBMCs were suspended in RPMI 1640 medium supplemented with 10% HS (5 × 10^5^/100 μl) and then were treated with alemtuzumab (10 μg/ml). Cells were incubated for 30 min at 37°C at different pH values, and necrosis was then evaluated by propidium iodide staining and flow cytometry. A representative experiment (n=5) is shown. **D** and **E.** Hemolytic assays of classical (D) and alternative (E) pathways of complement activation were performed at different values of pH as described under Materials and Methods. Data represent the mean ± SEM of 4-5 experiments performed in triplicate. * p<0.05 vs pH 7.3.

It is usually assumed that the destruction of IgG-coated target cells by complement is mediated through the activation of the classical pathway. However, studies published by Taylor's lab suggests a more complex picture, strongly dependent on the order in which the antibody and the complement source are added to target cells [36]. Working with B-cell lines treated with the anti-CD20 mAbs RTX or ofatumumab, the authors reported that when B cells were first opsonized with antibodies in serum-free medium, washed, and then cultured with HS, CDC is mediated through the activation of the alternative pathway of complement. By contrast, when anti-CD20 mAbs were added to B cells already suspended in HS, cytotoxicity is mainly mediated by the classical pathway of activation (36). Consistent with this unexpected observation, we found that when PBMCs were first opsonized with alemtuzumab in serum free medium, and then incubated with HS (10%), CDC was unaffected by the addition of Mg/EGTA, suggesting that cytotoxicity is mediated through the alternative pathway of activation. By contrast, when PBMCs were opsonized with alemtuzumab in the presence of HS (10%), CDC was strongly inhibited by Mg/EGTA, suggesting that cytotoxicity is mediated through the classical pathway of activation (Figure 3A). Interestingly, CDC assessed under both experimental conditions was similarly inhibited by low pH (Figures 3B and 3C), suggesting that acidosis prevents the activation of the alternative and classical complement pathways.

To further confirm that low pH inhibits both pathways, we analyzed the effect of pH on the ability of HS to destroy either IgG-coated SRBC or rabbit red blood cells suspended in medium supplemented with Mg-EGTA, the two hemolytic systems commonly used to evaluate the classical and alternative pathways of complement activation, respectively [37, 38]. We found that pH 5.5 and 6.0 markedly inhibited CDC against IgG-SRBC (Figure 3D). On the other hand, the lysis of rabbit red blood cells suspended in Mg-EGTA supplemented medium was shown to be significantly inhibited at pH 6.0 and nearly completely abrogated at pH 5.5 (Figure 3E). We conclude that low pH inhibits CDC mediated by both, the classical and alternative pathways of complement activation.

### Low pH impairs the generation of C3b, C4b and C3a induced by IgG-coated target cells

**Figure 4:**
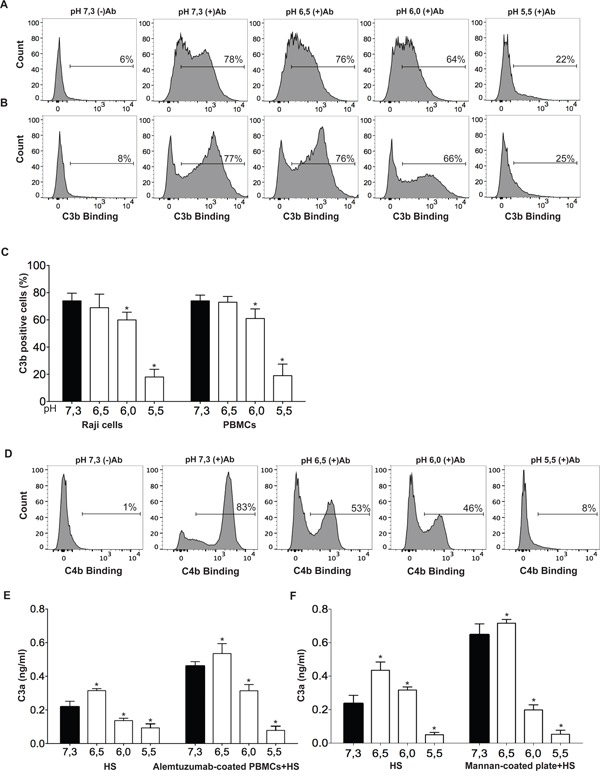
Low pH inhibits C3b, C4b and C3a generation **A-D.** RTX-treated Raji cells (5 × 10^5^/100 μl) and alemtuzumab-treated PBMCs (5 × 10^5^/100 μl) were incubated in the presence of 10% HS or 10% autologous HS, respectively, for 30 min at 37°C, at different pH values. Then, the amount of C3b deposited on Raji cells (A and C) and PBMCs (B and C) as well as the amount of C4b deposited on Raji cells (D) was determined using a FITC-labeled mAb directed to C3b or C4b and flow cytometry. Representative histograms and the mean ± SEM of 5-7 experiments are shown (* p<0.05 vs pH 7.3). **E.** HS (20%) was incubated in the absence or presence of alemtuzumab-treated PBMCs (5 × 10^5^/100 μl), for 30 min at 37°C, at different pH values. Then, the concentration of C3a in the culture medium was determined by ELISA. Values represent the mean ± SEM of 4-5 experiments performed in duplicate (* p<0.05 vs pH 7.3). **F.** HS (20%) was incubated on uncoated or mannan-coated plates for 30 min at 37ªC, at different pH values. Then, the concentration of C3a in the culture medium was determined by ELISA. Values represent the mean ± SEM of 4 experiments performed in duplicate (* p<0.05 vs pH 7.3).

To further analyze the mechanisms underlying the ability of acidic pH to prevent CDC, we first determined whether the inhibition of cytotoxicity correlated with a reduced deposition of C3b on the surface of target cells. In these experiments, we used Raji cells (Figure 4A and 4C) and PBMCs (Figure 4B and 4C) opsonized in serum free medium with RTX or alemtuzumab, respectively. Opsonized cells were incubated for 30 min at 37°C in the presence of 10% HS and the amount of C3b deposited on the cell surface was quantified by flow cytometry. Our results (Figure 4A-4C) show that low pH inhibited the generation/deposition of C3b on target cells. A similar inhibitory effect was observed when the deposition of C4b on alemtuzumab-coated PBMCs was examined (Figure 4D). Similar results were observed when PBMCs already suspended in 10% HS were opsonized with specific antibodies (data not shown).

We then looked at the effects of low pH on the production of C3a. In these experiments, autologous HS (20%) was incubated with or without (controls) alemtuzumab-coated PBMCs for 30 min, and the production of C3a was then evaluated by ELISA. The results obtained are showed in Figure 4E. As previously described, incubation of HS alone leads to the “spontaneous” activation of complement and the generation of low levels of C3a, perhaps reflecting the activation of the classical pathway induced by physiological concentrations of serum immune complexes [39]. Consistent with the ability of pH 6.5 to stimulate the alternative pathway of complement [40, 41], we found an enhanced generation of C3a at this pH value. By contrast, a significant reduction in the generation of C3a was observed when HS alone was incubated at pH 6.0 or 5.5. Working with alemtuzumab-coated PBMCs, on the other hand, we found that the production of C3a was significantly inhibited at pH 6.0 and 5.5 compared with pH 7.3. In fact, the ability of alemtuzumab-coated PBMCs to induce the production of C3a was completely abrogated at pH 5.5 (Figure 4E).

**Figure 5:**
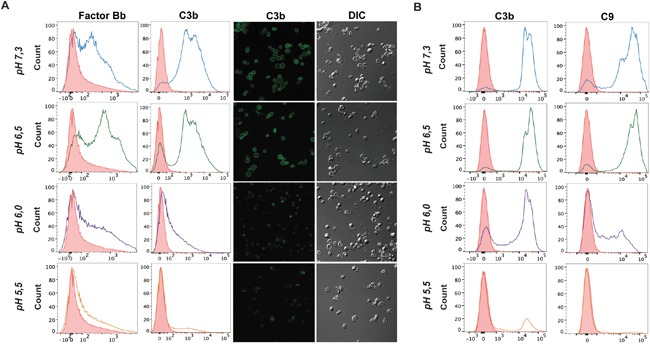
Low pH prevents C3b, factor Bb and C9 deposition on Candida albicans or Salmonella enterica **A.** *Candida albicans* (5 × 10^5^/200 μl) was incubated for 30 min in RPMI medium supplemented with untreated (open histograms) or heat-inactivated (filled histograms) HS (10%), adjusted to different pH values. Yeasts were then washed and the deposition of C3b and factor Bb was analyzed by flow cytometry or fluorescence microscopy. **B.** *Salmonella enterica serovar Enteritidis* was incubated for 30 min at 5 × 10^6^/200 μl in RPMI medium supplemented with untreated (open histograms) or heat-inactivated (filled histograms) HS (10%), adjusted to different pH values. Then C3b and C9 deposition on the bacterial surface was revealed by flow cytometry. Representative histograms or images from 4-7 experiments are shown.

We then looked at the influence of low pH on the lectin pathway of complement activation. To this aim, HS (20%) was incubated on mannan-coated plates for 30 min at 37°C and the production of C3a was then evaluated by ELISA. Results in Figure 4F show that pH values of 6.0 and 5.5 markedly prevented the enhancement in C3a generation induced by mannan. Low pH also strongly inhibited the deposition of the factor Bb and C3b on the surface of the fungi *Candida albicans* (Figure 5A), a strong inducer of the alternative pathway of complement [42], as well as the deposition of C3b and C9 on the surface of *Salmonella enterica* (Figure 5B). Overall, our observations suggest that the three major pathways of complement activation are inhibited by low pH and that this inhibition might compromise not only the anti-tumor activity mediated by therapeutic antibodies, but also the microbicidal action of the complement system.

### Inhibition of CDC by low pH is a reversible phenomenon and occurs in whole blood

**Figure 6:**
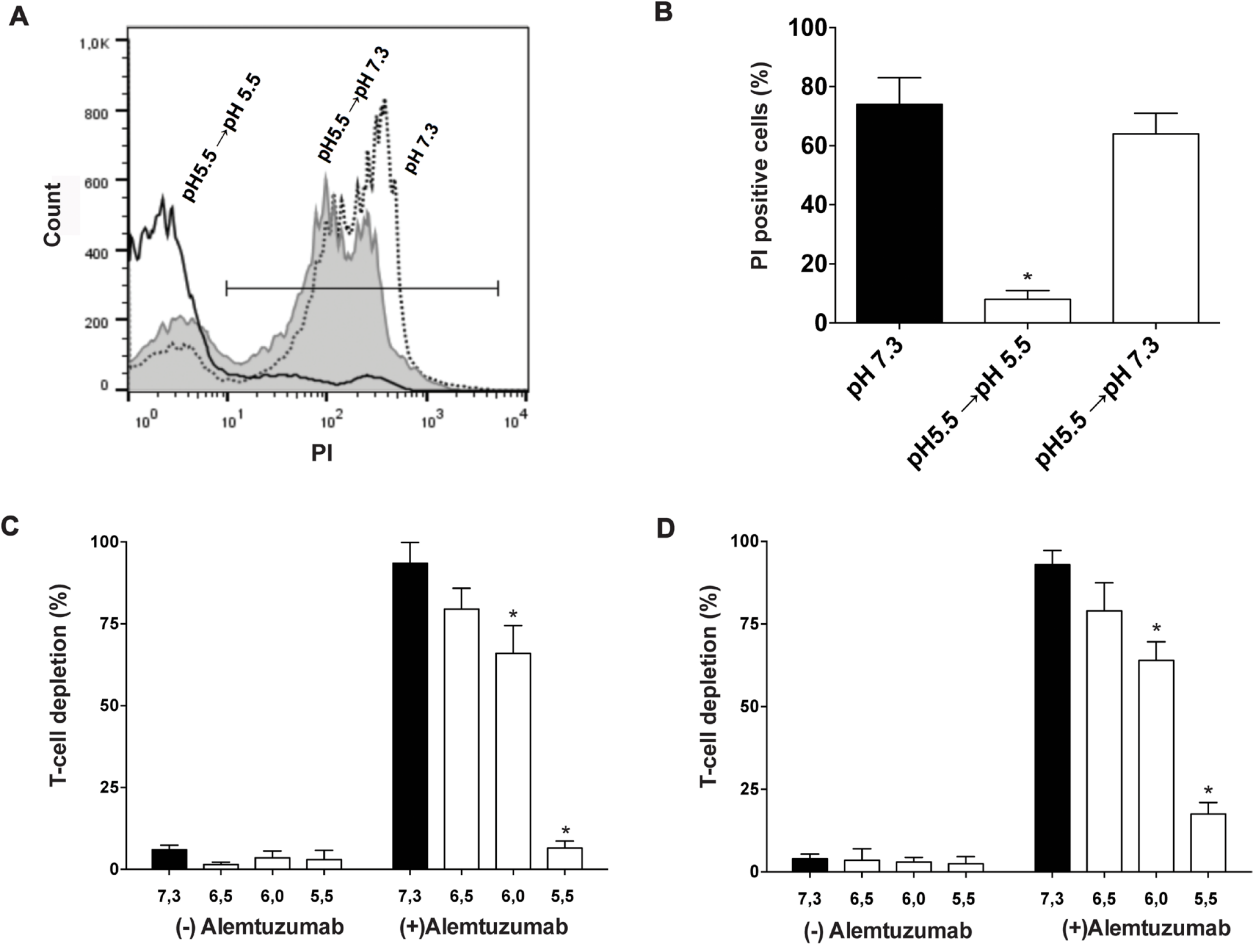
Inhibition of CDC by low pH occurs in whole blood and is a reversible phenomenon **A** and **B.** Alemtuzumab-treated PBMCs (5 × 10^5^/100 μl) were incubated for 30 min at 37°C with 10% autologous HS, at pH 5.5. Then, the pH was adjusted to pH 7.3 (grey histograms) or remained at pH 5.5 (black line), and cells were further incubated for a period of 30 min at 37°C. Finally, necrosis was evaluated by PI staining and flow cytometry. Controls represent alemtuzumab-treated PBMCs incubated for 60 min at 37°C and pH 7.3 in the presence of 10% autologous HS (dotted lines). Representative histograms or the mean ± SEM of 5 experiments are showed (*p<0.05 vs pH 7.3). **C** and **D.** Aliquots of peripheral blood (100 μl) collected on 3.2% sodium citrate (C) or bivalirudin (50μg/ml) (D) were adjusted to different pH values and treated with alemtuzumab (50 μg/ml). After 30 min incubation at 37°C, cells were stained with FITC-conjugated anti-CD3 mAb and PerCP-7AAD. The percentage of CD3^+^/7AAD^−^ cells was determined for each condition and the results were expressed as the percentage of T cell depletion compared with untreated blood samples. Controls were made by incubating aliquots of peripheral blood, adjusted to different pH values, without alemtuzumab. Data represent the mean ± SEM of 4-5 experiments. *p<0.05 vs pH 7.3.

Then, we analyzed whether the inhibition of CDC by low pH could be overcome upon pH neutralization. This was tested in a new set of experiments using alemtuzumab-coated PBMCs as target cells. Figures 6A and 6B show that abrogation of CDC by pH 5.5 was almost completely reversed upon pH neutralization, suggesting that inhibition of CDC by low pH is a reversible phenomenon. Finally, we addressed the question as to whether, in the more physiologic milieu of whole blood, low pH could also inhibit CDC. Whole blood assays were performed as previously described [43]. Preliminary experiments showed that consistent with the weak expression of CD52 by neutrophils [44], they were not susceptible to CDC induced by alemtuzumab in whole blood assays. By contrast, both T and B lymphocytes were shown to be highly susceptible to CDC (not shown). To evaluate the ability of low pH to suppress CDC, aliquots of whole blood (100 μl) collected on either 3.2% sodium citrate (Figure 6C) or the thrombin inhibitor bivalirudin (50 μg/ml) (Figure 6D), were adjusted to different pH values and treated with alemtuzumab (50 μg/ml) for 30 min at 37°C. Then, the absolute number of viable CD3+ T cells was determined by flow cytometry. In agreement with the observations made in isolated PBMCs, we found that low pH markedly inhibited T cell depletion in assays performed in whole blood collected in either sodium citrate or bivalirudin. In fact, T cell depletion mediated by complement was significantly inhibited at pH 6.0 and almost completely prevented at pH 5.5 (Figures 6C and 6D).

## DISCUSSION

Using HS as a complement source, we here showed that severe acidosis (pH values of 5.5 and 6.0) inhibits CDC against IgG-coated target cells. Almost identical results were observed using B-cell lines and normal PBMCs, suggesting that low pH inhibits CDC irrespective of the nature of target cells. Abrogation of CDC by low pH appears to be a reversible phenomenon since it was completely reversed upon pH neutralization. Our observations also indicate that extracellular acidosis is able to inhibit the three major pathways of complement activation and that this inhibition might compromise not only the anti-tumor activity mediated by therapeutic antibodies, but also the microbicidal action of the complement system which also take place at inflammatory environments usually characterized by low values of extracellular pH [1–6]. Interestingly, inhibition of CDC was observed not only using cell lines and isolated PBMCs as target cells, but also in the more physiologic milieu of whole blood, suggesting that low pH might compromise the function of complement not only *in vitro* but also *in vivo*.

Previous reports have analyzed the influence exerted by acidic values of pH on the alternative and lectin pathways of complement activation. Studies performed with isolated complement components have shown that the optimal pH for the initiation and amplification of the alternative pathway of complement is 6.4, perhaps reflecting an increased generation of both C3 and C5 convertases [40, 41, 45]. Acidic values of pH have also shown to modulate the ability of the acute phase reactant C-reactive protein (CRP) to activate the complement system. It is well known that CRP activates the classical pathway of complement upon reaction with phosphocholine-containing or polycation-containing ligands, and it has been shown that mildly acidic conditions (optimal pH ~ 6.3) enables CRP to effectively activate complement even in the absence of these ligands [46]. Moreover, Zhang and coworkers [47] have reported that mild acidosis (pH 6.5) favors the interaction of CRP with a second acute phase reactant, L-ficolin, leading to an enhanced deposition of C3 on the bacterial surface, and an increased bactericidal activity of human serum.

C1q is the key molecule required for the initiation of the classical pathway of complement activation. It interacts with the Cγ2 domain of IgG or the Cμ3 domain of IgM when these antibodies have recognized antigens, leading to the formation of immune complexes [48]. Previous studies have analyzed the influence exerted by low pH on the recognition of IgG by C1q, however, contrasting results have been reported. Using immobilized human IgG as an immune complex model, Kaul and Loos [49] have reported that the recognition of IgG by C1q is lower at pH 6.5, 6.0, and 5.5, compared with pH 7.4. By contrast, using heat-aggregated human IgG1, Roumenina and coworkers [50] demonstrated that the recognition of IgG by C1q shows an optimal pH of 5.5, and markedly decreases at neutral values of pH. These contrasting results suggest that the effect exerted by pH on the activation of the classical pathway of complement might be strongly influenced by the characteristics of the triggering stimulus. Not only the effector pathways of complement activation are susceptible to be modulated by pH in the range comprised between 5.0 and 7.5, but also natural complement inhibitors have shown to act in a pH-dependent manner. The ability of CR1 to promote the cleavage of C3b by factor I shows an optimum pH of 7.5 and decreases at pH values of 6.0 and 5.0. By contrast, the activity of factor H has been shown to be markedly higher at pH 5.0 compared to pH 7.5 [51] while the binding of factor I to either C3b or factor H reaches a maximum at pH 4.0 to 5.5, showing a sharp decrease at neutral pH values [52]. An enhanced ability of factor H to promote the cleavage of C3b by factor I might contribute to the inhibition of CDC by low pH observed in the present study.

The mechanism by which low pH impairs CDC is yet to be defined. The complement proteins C3 and C4 have an internal thioester which becomes available to react with amino or hydroxyl groups on the target surface upon activation [53]. This step plays a critical role in complement activation and appears to be strongly dependent on the presence of a single histidine residue (either in C3 or C4) which acts as a base or a nucleophile to attack the internal thioester, leading to the interaction of C3b and C4b with the target cell surface [54, 55]. Because the imidazole side chain of histidine has a pKa of 6.0, we hypothesize that protonation of this histidine residue at pH values of 6.0 and 5.5 might impair its ability to attack the internal thioester, thus preventing both, the binding of C3b and C4b to the target cell surface and the assembly of C3 convertases. Further experiments are needed to test this hypothesis.

Strikingly, no previous studies have investigated the influence exerted by extracellular pH on the lysis of IgG-coated cells by complement. Because the recognized ability of IgG-coated cells to activate the classical complement pathway, it has been assumed that the formation of the membrane attack complex (MAC) is mainly induced through the activation of the classical pathway and that the alternative pathway merely acts as a positive feedback amplification loop. This assumption is also consistent with the traditional view according to which the activation of the alternative pathway is considered to be antibody-independent. However, a number of studies have clearly shown that immune complexes can activate the alternative pathway of complement [56]. In line with these reports, and in agreement with previous observations made by Beum and coworkers in B cell lines opsonized with anti-CD20 antibodies [36], we found that the activation pathway responsible for CDC against alemtuzumab-opsonized PBMCs depends on the experimental conditions under which the assay is performed. When PBMCs were first opsonized with alemtuzumab in serum-free medium and then incubated with HS, CDC was shown to be unaffected by EGTA/Mg, suggesting that cytotoxicity is mediated through the activation of the alternative pathway. By contrast, when alemtuzumab was added to PBMCs already suspended in HS, CDC was abrogated by EGTA/Mg, suggesting the involvement of the classical pathway. The reasons underlying these differences are unclear, however, we found that pH values of 6.0 and 5.5 inhibited CDC assessed in both experimental conditions, reflecting the ability of severe acidosis to prevent the activation of the alternative and classical pathways of complement.

Cancer therapy based on the use of mAb is one of the most successful approaches for the treatment of patients with hematological malignancies and some solid tumors [57]. Mechanisms contributing to mAb-induced tumor cell death include CDC, antibody-dependent cellular cytotoxicity (ADCC), and induction of apoptosis by direct transmembrane signaling [57, 58]. A large body of evidence suggests that CDC plays an important role in the therapeutic activity of some mAb [59]. C1q deficient mice show a poor response to mAb-based therapies [60]. Patients with chronic lymphocytic leukemia suffer acute complement depletion upon administration of RTX, indicating an effective activation of complement *in vivo* [61]. Moreover, polymorphisms in complement components and complement regulatory factors have shown to be associated to the efficacy of immunotherapy [62, 63].

Our present results suggest that low values of extracellular pH, similar to those found in solid tumors [12–16], might represent an important obstacle for mAb-based therapies because CDC would be impaired. Importantly, not only CDC, but also ADCC mediated by NK cells against IgG-coated tumor cells appears to be strongly inhibited by low pH [26, 64], emphasizing that acidic microenvironments might limit the effectiveness of therapeutic mAb. This raises the question whether anti-tumor mAb could be combined with agents capable of raising tumor extracellular pH in order to improve the therapeutic effect. Interestingly, considering that acidic environments not only promotes tumor invasive growth and metastasis [17, 18], but also induces multi-drug resistance due to the neutralization of weak base chemotherapeutic drugs [19], it has been suggested that manipulation of the extracellular pH might have considerable potential as cancer therapy [65]. In fact, preclinical studies have shown that proton pump inhibitors increase tumor extracellular pH and exert anti-tumor effects [66–67]. Interestingly, not only proton pump inhibitors but also mTOR inhibitors such as rapamycin or rapamycin analogues (rapalogs), used in the treatment of cancer [68, 69] have shown to decrease lactate production by tumor cells increasing extracellular pH [70]. Whether raising tumor extracellular pH by either proton pump inhibitors or rapamycin could improve the efficacy of mAb-based therapies in cancer patients is yet to be examined.

## MATERIALS AND METHODS

### Cells

Peripheral blood mononuclear cells (PBMCs) were isolated from heparinized human blood samples by standard density gradient centrifugation on Ficoll-Hypaque, and resuspended in RPMI 1640 medium (Invitrogen Life Technologies) supplemented with the indicated concentration of autologous serum. The B cell lines Raji and Daudi were obtained from the American Type Culture Collection and cultured in RPMI 1640 medium supplemented with 10% heat-inactivated fetal calf serum, 50 U/ml penicillin, 50 μg/ml streptomycin, and 0.1 mM nonessential amino acids (Invitrogen Life Technologies).

### Culture conditions

Extracellular acidification was achieved by the addition of a precalculated volume of isotonic hydrogen chloride solution to either, the culture medium supplemented with HS or whole blood samples, as we had previously described [20]. In all cases, cell cultures were maintained at 37°C in a humidified atmosphere supplemented with 5% CO_2_, and the pH was checked by using a pH meter (Jensen Instrument Co).

### Complement-dependent cytotoxicity (CDC)

Unless otherwise stated, CDC assays were performed as follows. The B lymphoblast cell lines Raji and Daudi, and human PBMCs were treated with the indicated concentrations of the chimeric anti-CD20 mAb rituximab (RTX) or the humanized anti-CD52 mAb alemtuzumab, respectively. Then, cells (5 × 10^5^/100 μl) were incubated in RPMI 1640 medium supplemented with 10% human serum (HS), used as a complement source. Autologous HS was used in the assays performed with PBMCs. After 30 min of incubation at 37°C and different pH values, the percentage of necrotic cells was determined by propidium iodide (PI) staining and flow cytometry on a FACSCanto II instrument (BD Biosciences). In some experiments, CDC was assessed using cells suspended in Ca^2+^/Mg^2+^-supplemented veronal-buffered saline instead of RPMI medium. Moreover, CDC was also assessed using a colorimetric method to determine cell viability, instead of PI staining, based on the ability of metabolically active cells to reduce the tetrazolium compound [3-(4, 5-dimethylthiazol-2-yl)-5-(3-carboxymethoxyphenyl)-2-(4-sulfophenyl)-2H-tetrazolium] to a formazan product (CellTiter 96 aqueous one solution cell proliferation assay kit, Promega).

In all cases, inactivation of HS was induced by heating at 56°C for 30 min. When indicated the CDC assay was performed as follows: PBMCs were first suspended in RPMI 1640 medium supplemented with 10% HS (5 × 10^5^/100 μl), and the anti-CD52 mAb alemtuzumab was then added, in the absence or presence of MgCl_2_ (5mM) and EGTA (10 mM) (Mg-EGTA). Cells were incubated for 30 min at 37°C at different pH values and the percentage of necrotic cells was determined by propidium iodide (PI) staining and flow cytometry. To analyze whether the inhibitory effect induced by low pH on CDC was reversible, alemtuzumab-coated target cells (5 × 10^5^/100 μl) were first incubated with RPMI 1640 medium supplemented with 10% HS for 30 min at pH 5.5. Then, the pH of the culture medium was neutralized by the addition of isotonic NaOH, and cells were further incubated for 30 min at pH 7.3. After this time, necrosis was evaluated as described above. Controls were carried out by incubating alemtuzumab-coated target cells for 60 min at pH 7.3 or 5.5.

### Hemolytic assays of classical and alternative pathways of complement activation

Assays were performed as previously described with minor modifications [37, 38]. Briefly, hemolytic assay for evaluation of the classical pathway of complement activation was performed using 5 × 10^7^ sheep red blood cells (SRBC) opsonized with rabbit IgG antibodies anti-SRBC (Sigma-Aldrich). Cells were suspended in RPMI 1640 medium without phenol red supplemented with 10% HS and adjusted to different pH values, in a final volume of 100 μl. Cells were then incubated for 30 min at 37°C. Then, cells were centrifuged and hemolysis was evaluated by measuring OD at 540 nm. Blanks were prepared with 5 × 10^7^ SRBC opsonized with rabbit IgG antibodies anti-SRBC, suspended in RPMI 1640 medium without phenol red supplemented with 10% heat-inactivated HS and adjusted to different pH values, in a final volume of 100 μl. The OD of 5 × 10^7^ SRBC lysed by the addition of 100 μl of distilled water was taken as 100% lysis. In all cases, the absorbance of blank tubes was lower than 10% compared with the absorbance of the tubes with 100% lysis. The percentage of lysis for each experimental condition was calculated as follows: [OD_540_ (X) – OD_540_ (blank)] / [OD_540_ (100%) – OD_540_ (blank)] / x 100. The hemolytic assay for evaluation of the alternative pathway of complement activation was performed using 2.5 × 10^7^ rabbit red blood cells suspended in RPMI 1640 medium without phenol red supplemented with 20% HS, 5mM MgCl_2_ and 10 mM EGTA (Mg/EGTA) adjusted to different pH values, in a final volume of 100 μl. Cells were incubated for 30 min at 37°C, centrifuged, and hemolysis was then evaluated by measuring OD at 540 nm. The percentage of lysis for each experimental condition was calculated as described above.

### Analysis of C3b and C4b deposition on the surface of IgG-coated target cells

Raji cells and PBMCs were treated with the anti-CD20 mAb RTX (2μg/ml) or the anti-CD52 mAb alemtuzumab (10 μg/ml), respectively, for 15 min at 37°C. Then, cells (5 × 10^5^/100 μl) were incubated for 30 min at 37°C in RPMI 1640 medium supplemented with 10% of HS, at different values of pH. After washing, C3b and/or C4b deposition on the cell surface was revealed by flow cytometry using specific FITC-labeled mAbs (AssayPro).

### Analysis of C3b, factor Bb, and C9 deposition on the surface of *Candida albicans or Salmonella enterica*

*Candida albicans* (ATCC10231) was incubated for 30 min at a density of 5 × 10^5^/200 μl in RPMI medium supplemented with 10% HS, adjusted to different pH values. After washing, C3b and factor Bb deposition on the yeast surface was revealed by flow cytometry using specific FITC- or APC-labeled mAbs (AssayPro). *Salmonella enterica serovar Enteritidis* (#5694) was incubated for 30 min at a density of 5 × 10^6^/200 μl in RPMI supplemented with 10% HS, adjusted to different pH values. After washing, C3b and C9 deposition on the bacterial surface was revealed by flow cytometry using specific FITC- or APC-labeled mAbs (AssayPro).

### Analysis of C3a generation induced by IgG-coated cells and mannan-coated plates

HS (20% in RPMI 1640 medium) was incubated for 30 min at 37°C with or without PBMCs (5 × 10^5^/100 μl), previously treated with the anti-CD52 mAb alemtuzumab (10 μg/ml). Then, the presence of C3a in the culture medium was analyzed by ELISA (Quidel).

To analyze the influence of low pH on the lectin pathway of complement activation we used mannan-coated plates, as previously described [71]. Briefly, HS (20%) was incubated for 30 min at 37°C in 96 well Nunc Maxisorb microtiter plates, uncoated or coated with 10 μg/ml of mannan (Sigma-Aldrich). Then, the production of C3a was analyzed by ELISA.

### Measurement of complement-dependent cytotoxicity in whole blood

It was performed as previously described [42]. Unmanipulated peripheral blood of healthy donors collected on either 3.2% sodium citrate or the specific thrombin inhibitor bivalirudin (Amuprux, Lab. Raffo, Argentina) (50μg/ml), were adjusted to different pH values and treated with alemtuzumab, to a final concentration of 50 μg/ml. Whole blood samples were incubated for 30 min at 37°C, and then stained for 15 min at room temperature with FITC-conjugated anti-CD3 mAb and PerCP-7AAD (BD Biosciences). Samples were then lysed with a whole blood lysing solution (BD Biosciences) to eliminate red blood cells and analyzed by flow cytometry. The percentage of CD3^+^/7AAD^−^ cells was evaluated for each condition and the results were expressed as the percentage of T cell depletion compared with untreated blood samples. Controls were made by incubating aliquots of peripheral blood adjusted to different pH values without alemtuzumab.

### Statistical analysis

Student's paired t test was used to determine the significance of differences between mean values, and p<0.05 was determined to indicate statistical significance.
